# External Ventricular Drainage: A Practical Guide for Neuro-Anesthesiologists

**DOI:** 10.3390/clinpract13010020

**Published:** 2023-01-31

**Authors:** Alessandro Bertuccio, Stefano Marasco, Yaroslava Longhitano, Tatsiana Romenskaya, Angela Elia, Gianluca Mezzini, Matteo Vitali, Christian Zanza, Andrea Barbanera

**Affiliations:** 1 Department of Neurosurgery, AON SS. Antonio e Biagio e Cesare Arrigo University Hospital, 15121 Alessandria, Italy; 2 Department of Neurosurgery, IRCCS S. Matteo University Hospital, University of Pavia, 27100 Pavia, Italy; 3 Department of Anesthesiology and Perioperative Medicine, University of Pittsburgh, Pittsburgh, PA 15261, USA; 4 Department of Physiology and Pharmacology, Sapienza University of Rome, P.le A. Moro 5, 00185 Rome, Italy; 5 Foundation “Ospedale Alba-Bra”—Department of Emergency Medicine, Anesthesia and Critical Care Medicine, Michele and Pietro Ferrero Hospital, 12060 Verduno, Italy

**Keywords:** intracranial pressure, subarachnoid hemorrhage, traumatic brain injury, intraventricular hemorrhage, external ventricular drainage, cerebrospinal shunt, external lumbar drainage, intracranial pressure monitoring

## Abstract

External ventricular drainage is often considered a life-saving treatment in acute hydrocephalus. Given the large number of discussion points, the ideal management of EVD has not been completely clarified. The objective of this study was to review the most relevant scientific evidence about the management of EVD in its main clinical scenarios. We reviewed the most recent and relevant articles about indications, timing, management, and complications of EVD in neurocritical care, with particular interest in patients with subarachnoid hemorrhage (SAH), severe traumatic brain injury (TBI), and intraventricular hemorrhage (IVH) using the following keywords alone or matching with one another: intracranial pressure, subarachnoid hemorrhage, traumatic brain injury, intraventricular hemorrhage, external ventricular drainage, cerebrospinal shunt, intracranial pressure monitoring, and ventriculoperitoneal shunt. In the management of EVD in SAH, the intermittent drainage strategy is burdened with an elevated risk of complications (e.g., clogged catheter, hemorrhage, and need for replacement). There seems to be more ventriculoperitoneal shunt dependency in rapid weaning approach-managed patients than in those treated with the gradual weaning approach. Although there is no evidence in favor of either strategy, it is conventionally accepted to adopt a continuous drainage approach in TBI patients. Less scientific evidence is available in the literature regarding the management of EVD in patients with severe TBI and intraparenchymal/intraventricular hemorrhage. EVD placement is a necessary treatment in several clinical scenarios. However, further randomized clinical trials are needed to clarify precisely how EVD should be managed in different clinical scenarios.

## 1. Introduction

External ventricular drainage (EVD) is one of the most commonly performed surgical procedures in neurosurgery. Described for the first time in 1744 by French surgeon Claude Nicholas Le Cat [1] (Srinivasan et al. 2014), it is often considered a life-saving treatment in cases of acute hydrocephalus due to a variety of pathologies and/or in cases of elevated intracranial hypertension. The presence of a computerized transduction system makes it possible to monitor intracranial pressure (ICP) continuously, even if it is associated with a higher risk of complications when compared with intraparenchymal monitoring devices [2] (Bales et al. 2019). The temporary need for outward shunting of cerebrospinal fluid during brain infections may be another indication for EVD [3] (Bratton 2007). However, despite the quick and relative simplicity of the surgical procedure, it is not free from the risk of complications, such as infections (e.g., meningitis and ventriculitis), hemorrhage along the trajectory, and malposition or obstruction of the catheter itself [4].

Established management practices for moderate and severe traumatic brain injury (TBI) are centred on minimising secondary injury through normalising intracranial homeostasis. This is achieved through a therapeutic paradigm principally centred on the avoidance of raised intracranial pressure (ICP) and maintenance of cerebral perfusion pressure (CPP). Contemporary ICP management strategies utilize a sequential escalation of therapeutic intensity until ICP control is achieved, with protocols based on the Brain Trauma Foundation (BTF) guidelines [1]. Initial medical treatments for intracranial hypertension include sedation, mild hypocapnia, and hyperosmolar therapy. When intracranial hypertension is refractory to these interventions, therapies including diversion of cerebrospinal fluid (CSF), barbiturate coma, and decompressive craniectomy can be considered. [5].

Despite being one of the most widespread neurosurgical procedures, there is great variability in current technical and management practices, and the proper management of EVD remains controversial. Variability is not only related to the surgical technique and postoperative complications but also the frequency, location, use of impregnated EVD catheters, and, above all, weaning and drainage strategies. 

However, the management with CSF drainage in patients with severe TBI remains a debated topic. There is a larger evidence base on this topic examining the use of external ventricular drainage (EVD) in this type of patients, with significantly less evidence examining external lumbar drainage (ELD) as a possible treatment to reduce ICP. The lack of literature on the topic of ELD in this regard is probably due to the circumstance that CSF-controlled lumbar drainage was considered a contraindication in patients with increased ICP because of the possibility of transtentorial or tonsillar hernia. Over the past decades, studies have indicated that it could be a potential treatment when used on accurately selected patients. At the present time, ELD, unfortunately, is a method that is not included in the current BTF guidelines due to a poor scientific evidence.

The aim of this article was to review the literature and to present a practical guide on EVD management based upon the most recent recommendations.

## 2. Methods

The literature search was performed using the PubMed/MEDLINE (https://pubmed.ncbi.nlm.nih.gov accessed on 1 November 2022) search engine, in addition to Medical Subject Headings (MeSH) terms. “External Ventricular Drain”, “Intracranial Pressure”, “ICP Monitoring”, “Subarachnoid Hemorrhage”, “Intraventricular Hemorrhage”, “External lumbar drainage”, “Cerebral Herniation”, “Traumatic Brain Injury”, “Non-invasive ICP Monitoring”, and “Acute Hydrocephalus” were the MeSH terms implemented. Our review considered the latest (period from 2010 to 2022) relevant articles and focused on the indications, timing, management, and complications of EVD in neurocritical care, with particular interest in patients with subarachnoid hemorrhage (SAH), severe traumatic brain injury (TBI), and intraventricular hemorrhage (IVH). Pediatric cases were excluded. No restrictions were placed on the language of the articles. Finally, 53 articles were considered in our selection.

## 3. Discussion

### 3.1. Overview

**Figure 1:**
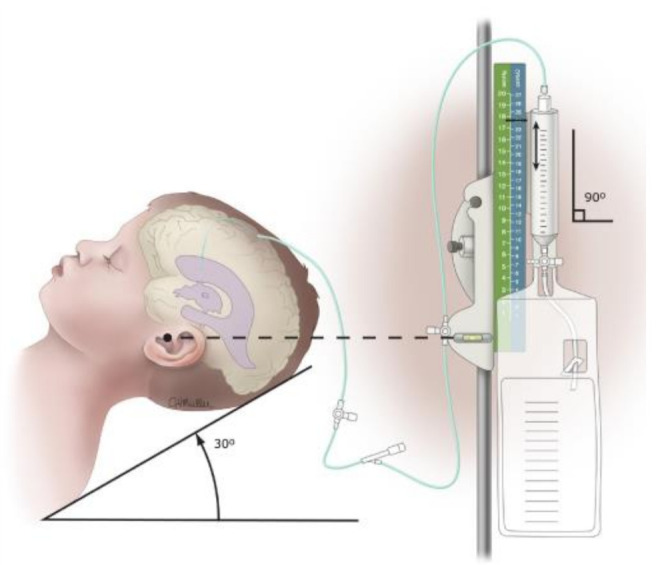
External ventricular drain (EVD).

The main indication for EVD is acute hydrocephalus, which could be related to different pathologies, such as SAH, intraparenchymal hemorrhage (with or without intraventricular involvement), infections, brain tumors, and shunt failure. Additionally, the Brain Trauma Foundation guidelines for the management of severe TBI include EVD (see Figure 1) as a surgical treatment and for monitoring ICP [6].

One of the first topics discussed is the most suitable environment to perform EVD placement. Often in an emergency, many centers perform EVD in places other than the operating room (OR), such as the emergency room (ER) or intensive care unit (ICU). Several studies, many retrospective, have aimed to compare the rate of complications in patients who have undergone EVD positioning in the OR vs. patients whose procedures were performed in the ICU or ER [7,8,9,10] (Schodel 2012, Foreman 2014, Altschul 2020, Dawod 2020). In particular, Schodel et al. examined 312 cases and compared the complication rates (e.g., CSF infection and postprocedural hemorrhage along the trajectory of the catheter) in patients who underwent EVD conventionally by mechanical drilling in the OR with patients whose EVD was positioned with a Bolt Kit System (BKS) in the ICU. The results showed that the postprocedural rate of infection and bleeding along the catheter trajectory was significantly lower in the BKS group (*p* = 0.032 and *p* = 0.018, respectively) [7] (Schodel 2012). In a single stroke center, Altschul et al. retrospectively analyzed the infection rate in 710 patients who underwent EVD placement. The infection rate was 13% for ER EVD, compared with 7.7% for ICU/OR EVD (*p* = 0.03) [9] (Altschul 2020). Finally, in a recent systematic review, the accuracy in positioning of the EVD, according to the Kakarla grading score, was better when performed in the OR than in the ICU (84.25% vs. 68.29%). The rate of iatrogenic bleeding was slightly higher in ICU EVD vs. OR EVD (18.16% vs. 17.50%). The rate of ventriculostomy-related infections was slightly lower when performed in the ICU than in the OR (7.28% vs. 8.06%), suggesting that there are likely specific patient populations that would benefit from EVD placement in the ICU vs. the OR setting [10]. However, it is important to note that one-shot antibiotic prophylaxis is always performed when an EVD is placed in the OR, which is not always the case for EVD placement in the ICU, so there is a lower risk of infection in OR-EVD placement [11]. As an emergency and rapid surgical intervention, different environments are used for EVD placement. In most neurosurgery units, EVD is applied in the OR; however, the ICU or, in exceptional cases, a general ward have been reported as alternative environments [12]. Notably, EVD placement outside an OR has been related to a higher risk of postoperative infections [13,14]. Therefore, the use of an OR as the preferred environment is generally accepted.

Several materials are used for EVD catheters. The main difference is between metal needles and polyurethane catheters. Metal needles are smaller (1.2 mm) and require a smaller skin incision, polyurethane catheters are larger (3 mm) and are integrated with a tip sensor for ICP monitoring. Considering its advantages, polyurethane EVD is routinely used in most hospitals, especially in cases of IVH or TBI. However, when ICP monitoring is not necessary or there is no ventricular hemorrhage, the metal needle may be a satisfactory alternative. Additionally, EVD catheters can be impregned with antibiotics (e.g., clindamycin, rifampicin, or gentamycin) or coated with antimicrobial materials (e.g., silver or hydrogel). Both have been related to a lower incidence of postoperative infections compared to uncoated EVD, without a significant difference between antibiotic-impregnated or coated catheters [15,16]. Therefore, the employment of impregnated or coated catheters is observed similarly, although there is a slight preference for silver-coated EVD [12].

### 3.2. EVD in Subarachnoid Hemorrhage 

Subarachnoid hemorrhage (SAH) is a common cerebrovascular disorder with high potential for morbidity and mortality [17]. In a conspicuous percentage of cases, patients with SAH have been shown to develop acute obstructive hydrocephalus, which is treated with EVD placement [18]. Most authors agree to keep cerebrospinal fluid (CSF) drainage low, compatibly with the gravity of the hydrocephalus and ICP value, setting the EVD system very high or closed, since there is historical scientific evidence showing that there is an elevated risk of aneurysmal re-breakage with excessive ICP reduction [19].

#### 3.2.1. Intermittent vs. Continuous Drainage 

In the literature, the two main accepted strategies for managing CSF drainage are continuous and intermittent. Intermittent EVD drainage is an “on demand” draining strategy. The EVD remains closed and is opened to drain only when the patient becomes symptomatic or ICP increases. In detail, for patients in the ICU, CSF release is suggested when ICP > 20 mm Hg is recorded for more than 5 min, and it is clamped when a normal ICP value is reached [16,18]. In patients who are awake and in whom a neurological assessment is possible, symptoms should determine proper EVD management. Headache, nausea or vomiting, and CSF leakage from the wound are indications to open EVD drainage. Despite the need for continuous monitoring, intermittent EVD drainage did not require further clinical concerns. In continuous EVD drainage, the drain is left open. However, this strategy is associated with more clinical questions about how much to drain, when and how to wean the EVD, and whether intermittent ICP monitoring is required. The volume of CSF to be released is set by the physician. Classically, EVD is set to 10 mL/h, aiming to drain approximately 100–300 mL per day [13]. Then, EVD drainage is adjusted in relation to neuroimaging, underlying pathology, and ICP monitoring. Theoretically, in cases of SAH, continuous EVD drainage allows better clearance of CSF, so it could reduce the incidence of vasospasm, despite a higher risk of ventricular collapse [4,20]. Comparing continuous vs. intermittent EVD approaches, no significant differences have been reported in relation to incidence of vasospasm, ventriculoperitoneal shunt (VPS) dependency, and length of hospital stay [4,21]. In a randomized clinical trial, Olson et al. reported a higher rate of complications (e.g., clogged catheter, EVD replacement, and hemorrhage) in an intermittent CSF drainage group than in a continuous drainage group, but no significant differences were found with regard to the risk of vasospasm [22].

#### 3.2.2. Rapid vs. Gradual Weaning Strategy

A further topic of discussion concerns weaning strategies, which can be divided into rapid vs. gradual EVD weaning. Although continuous drainage is related to rapid weaning, and intermittent drainage is related to more gradual weaning, these concepts remain distinct.

The rapid wean approach (RWA) consists of immediate closure of the system. Based on neuroimaging and clinical aspects, neurosurgeons and neurointensivists estimate a favorable period to attempt discontinuation of the EVD. The drainage is then stopped, and the catheter is used for monitoring purposes only. Ultimately, symptomatic hydrocephalus can be both safely diagnosed and rapidly treated. The theory underlying RWA is that a rapid increase in CSF pressure following the closure of the EVD could induce the recruitment of CSF resorption pathways in arachnoid granulation [23]. Ultimately, RWA potentially increases the probability of earlier and successful discontinuation of the EVD.

Alternatively, the gradual wean approach (GWA) requires a more prolonged period of EVD discontinuation. The drain is left open and gradually raised over several days. Usually, when the drain system is set above 25 cm H_2_O, it is then clamped and discontinued. The theoretical rationale is that a gradual increase in CSF pressure over 2–3 days could lead to progressive rebalancing between production and reabsorption pathways [24].

Different data have been observed comparing EVD wean strategies. In 2004, Klopfenstein et al. assessed the relationship between EVD wean strategy and the rate of VPS and length of hospital stay in 81 patients with subarachnoid aneurysmal hemorrhage [25]. The results did not show any significant relationship between EVD wean approach and VPS placement rate (63.4% in RWA vs. 62.5% in GWA; *p* = 0.932). However, RWA was related to a shorter hospital stay (mean days: 15.8 days in GWA vs. 12.7 days in RWA, *p* < 0.001) [26]. Recently, a multicenter observational study compared the two EVD weaning approaches and their relationships with VPS placement and length of hospital stay [25]. The study involved a cohort of 185 patients with subarachnoid aneurysmal hemorrhages that required EVD placement. Data analysis proved that RWA was associated with a lower rate of VPS at 90 days (VPS-free: 83% in RWA vs. 69% in GWA, HR: 0.52, *p* = 0.041). Similarly, the number of wean attempts was directly proportional to a greater likelihood of VPS (95% CI: 1.8–4.8, OR = 2.9, *p* < 0.0001). Conversely, no statistically significant difference was found between the two weaning strategies and length of hospital stay [24]. In 2018, a German study enrolled and randomized 965 patients with SAH and EVD into GWA and RWA groups [27]. The results showed a higher and earlier rate of VPS placement in RWA than in GWA (93.9% vs. 61.2%, respectively). Accordingly, statistical analysis proved an independent association between RWA and shunt dependency (*p* = 0.026) [27].

Lastly, in a recent meta-analysis, RWA was associated with a shorter hospital stay (SMD = 0.34, 95% CI: 0.22–0.47, *I*-squared = 0%), while no difference was observed in terms of VPS dependency (RR = 1.24, 95% CI: 0.99–1.55, *I*-squared = 34.6%) [4]. A further systematic review with meta-analysis aiming to study the effects of EVD wean strategies is ongoing [28].

### 3.3. EVD in Severe TBI

In TBI, EVD plays a role in ICP monitoring and CSF shunting in cases of elevated ICP. To date, no studies examining the proper management of EVD in TBI were found. In a recent prospective observational study that aimed to compare intraparenchymal vs. EVD ICP monitoring, intermittent drainage was used [20]. Interestingly, the results confirmed the therapeutic benefit of intermittent EVD drainage, reporting both fewer episodes of refractory ICP (*p* > 0.001) and better survival (*p* = 0.006) [20].

The Brain Trauma Foundation defined the dual roles of EVD in the management of severe TBI as both therapeutic and as a monitoring tool. Continuous EVD drainage was recommended more than intermittent EVD drainage (Level III) [29].

The literature lacks high-quality evidence supporting the use of intermittent vs. continuous EVD drainage [29]. Ultimately, the most common worldwide tendency is to use a continuous CSF draining system [29,30].

### 3.4. EVD in Intraparenchymal Hemorrhage with Intraventricular Involvement

Intraparenchymal/intraventricular hemorrhage (IH/IVH) is another cause of CSF flow obstruction and acute obstructive hydrocephalus. EVD placement is required when medical management is not sufficient to reduce ICP, and clinical deterioration occurs. Early EVD placement in IH/IVH patients in good neurological condition is controversial. It is well known that EVD reduces ICP in patients with IH/IVH; however there are no statistically significant data about the reduction of morbidity and mortality [31]. Although no randomized clinical trials are available, some observational studies have described better outcomes with intraventricular fibrinolytic treatment in some patients with associated SAH and IH/IVH [32,33]. A phase II clinical study recently tested the safety of using intraventricular thrombolytics in cases of massive intraventricular bleeding. In particular, the use of recombinant plasminogen factor (rt-PA) was tested, and it was found that a dosage of 1 mg every 8 h ensured good liquor clearance in the absence of an elevated risk of bleeding [34]. A phase III study to analyze the impact on clinical outcome is ongoing (CLEAR III Clot Lysis: Evaluating Accelerated Resolution of Intraventricular Hemorrhage Phase III).

### 3.5. Surgical Technique

Historically, several different entry points and EVD trajectories have been proposed [35]. Dandy’s and Keen’s points are two different techniques that aim to place the entry point in a more posterior and peritrigonal fashion. However, these trajectories are more prone to visual pathway damage, so they are not routinely performed. Similarly, Frazier’s technique requires an occipital–parietal trajectory, and it is usually recommended for pediatric patients or in cases of emergency posterior fossa procedures.

The most common technique is still the placement of the EVD catheter in the frontal region at Kocher’s point, aiming to reach the third ventricle. The procedure is easy, quick, and has a short learning curve. 

The patient is in the supine position with an elevation of 30–45 degrees and with the head in a neutral position to avoid significant distortion of anatomical landmarks. The entry point for placing the EVD is called Kocher’s point, which is located approximately 12 cm from the nasion (1 cm anterior to the coronal suture) and 3 cm lateral from the midline (along the midpapillary line).

**Figure 2:**
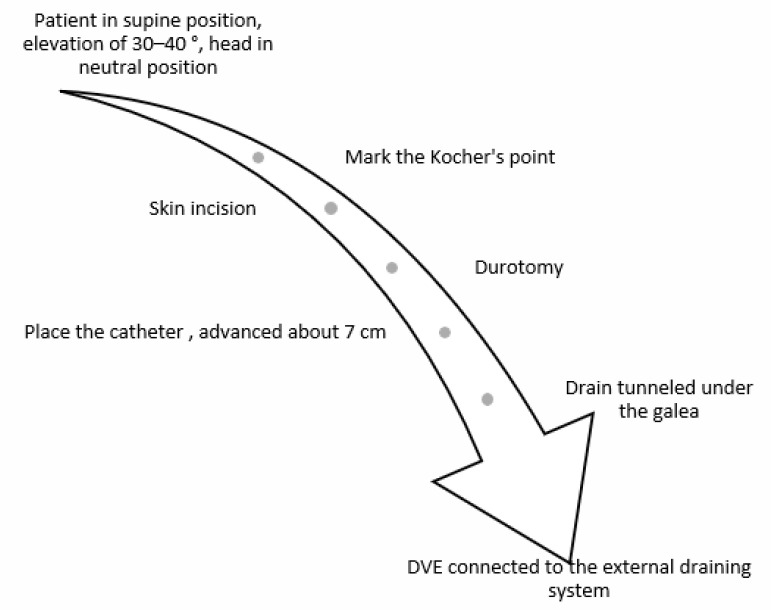
Algorithm for implantation of an EVD.

After skin incision, a bur hole is performed at Kocher’s point, then, after durotomy, the catheter is placed. The trajectory is directed toward the medial cantus on the coronal plane and about 1 cm anterior to the tragus on the sagittal plane. The catheter is advanced about 7 cm. The aim is to place the catheter at the foramen of Monro. Finally, the drain is tunneled under the galea and connected to the external drainage system (see Figure 2).

### 3.6. Complications

One of the most frequent complications is EVD blockage, which requires rapid replacement to avoid acute hydrocephalus. A major incidence of EVD failure has been related to the continuous EVD drain approach (RR = 0.45, 95% CI: 0.27–0.74, *I*-squared = 0%) [16].

Independent from any EVD management, other common complications are hemorrhage and catheter misplacement, with a reported incidence of up to 12% [36,37,38]. Factors affecting hemorrhagic complications include the use of oral anticoagulants or antiplatelet therapy, while brain edema and midline shift were the main risk factors for EVD misplacement [37].

One of the most serious EVD-related complications is infection, with possible progression toward ventriculitis or cerebritis. The incidence ranges from 0 to 27%, and it is primarily related to the duration of EVD [36]. Significant differences were observed when comparing infection rates between EVD approaches. Data from a recent meta-analysis proved that continuous EVD drainage is a risk factor (RR = 0.20; 95% CI: 0.05–0.72; *I*-squared = 0%) [16]. Conversely, no significant differences were reported when comparing EVD wean approaches (RR = 0.56, 95% CI: 0.06–3.93, *I*-squared = 73.2%) [16].

In 2015, a meta-analysis of studies on the incidence of infections following EVD placement was published. It revealed that the overall incidence of infection was 10.6/1000 catheter-days. It also showed that in patients with an EVD in place for less than 7 days, the incidence of infection was approximately 19.6/1000 catheter-days compared to an incidence of 8/1000 catheter-days when the EVD was in place for more than 10 days [39].

Thereafter, a prospective multicenter cohort study on EVD infections in the UK and Ireland was published. It highlighted a 9.3% rate of EVD infections at 30 days after placement. According to regression analysis, the duration of the EVD placement surgical procedure and frequent CSF sampling were independently associated with an increased risk of ventriculitis, regardless of whether the catheter was impregnated with antibiotics (i.e., silver). Furthermore, the same study showed that of all EVD catheters placed, 129 catheters were never sampled and were never associated with catheter infection/ventriculitis [40].

### 3.7. External Lumbar Drainage (ELD) for Refractory Intracranial Hypertension in Traumatic Brain Injury

The use of ELD for the treatment of ICP post-TBI is less common due to the potential risk of iatrogenic transtentorial hernia. In other ICP settings, however, the use of ELD has been successfully adopted, including in bacterial and cryptococcal meningitis and subarachnoid haemorrhage. A comparable success of ICP control as of ventriculostomy drainage is obtained with ELD with no significant complication rates, although evidence is insufficient to support ELD as an option in ICP control after TBI according to consensus guidelines [41,42,43,44].

Lumbar drainage represents a potential alternative means of CSF diversion to ventriculostomy because it avoids the passage of a drain through cerebral parenchyma. Insertion of a lumbar drain may be a technically and logistically simpler procedure, particularly in patients with isolated TBI and small lateral ventricles that make EVD insertion difficult or not feasible. Although it is believed that it may also control intracranial pressure, the safety concerns regarding lumbar drainage lie in the possible complication of cerebral herniation, with associated morbidity and mortality. to help guide current practice and future research directions, a synthesis of the safety and efficacy of external lumbar drainage for ICP control in TBI is needed, with reference to the safety and efficacy of the other available CSF diversion modality, external ventricular drainage [5].

Abdal-Centallas et al., Levy et al., Murad et al. in their studies reported a marked reduction in ICP after CSF diversion with an ELD. 

The three studies reporting the outcome of statistical tests of this effect all reported a statistically significant reduction in ICP after the introduction of lumbar drainage. Of four studies presenting data or observations on the effect of lumbar drainage on CPP, all reported a positive effect. Four studies presented observations on the effect of lumbar drainage on treatment intensity or requirement for other ICP-lowering therapies. 

All four reported a beneficial effect on the requirement of other therapies after the institution of lumbar drainage [45,46,47,48,49,50,51].

For the safety of ELD The available data identified by this systematic review is insufficient to draw firm conclusions on the safety profile of ELD in the management of raised ICP in TBI; however, the included studies here have not reported incidents of the usage of ELD directly contributing to mortality. A single case of ELD usage has been reported to result in pupillary changes requiring urgent surgery with good neurological recovery). However, the absence of significant complications in a series of 159 patients with TBI is unlikely to generate sufficient confidence to assuage the reservations held by some neurosurgical centers. In the single reported case of cerebral herniation requiring surgery, this was urgently recognized and appropriately managed, resulting in good neurological recovery. Despite historical concerns of “coning” from lumbar puncture or drainage, the results suggest that in the modern intensive care unit, there is sufficient clinical monitoring to facilitate early recognition of cerebral herniation through clinical and radiological assessment and prompt remedial action. Whilst a high mortality rate is not justification for therapeutics with the potential for harm, the alternative to lumbar drain is typically insertion of EVD; where this fails, decompressive craniectomy is typically indicated, and neither can be considered to be low morbidity. Although not reliable, the results reported here are suggestive of a low to very low risk of severe morbidity or mortality due to ELD in selected patients. If the surgical risk is considered, ELD offers the potential advantage of achieving ICP control without the need for EVD or decompressive craniectomy. the infection rates of the included studies were 3.9%, though some incidences of infection were in patients with both EVD and ELD. This incidence is similar to that reported in the literature, with infection rates of ELD reported as 4.2–7% [39,40] and EVD reported as 7-9% [52,53,54].

## 4. Conclusions

EVD placement is a life-saving surgical procedure in cases of acute hydrocephalus. In the management of EVD in SAH, the intermittent drainage strategy is burdened with an elevated risk of complications (e.g., clogged catheter, hemorrhage, and need for replacement). There seems to be more VPS dependency in RWA-managed patients than in those treated with GWA [26]. Although there is no evidence in favor of either strategy, it is conventionally accepted to adopt the continuous drainage approach in TBI patients. The actual benefits of intraventricular thrombolytic therapy in patients with IH/IVH are still under study. Further randomized clinical trials are needed to clarify the exact management of EVD in different clinical situations.

For the ELD, the literature appears to support the efficacy of lumbar drainage in ICP control where medical management has failed, though the data on its safety is encouraging but insufficient to draw conclusive recommendations. The efficacy and safety profile of ELD in comparison to EVD is not known, though ELD appears to be beneficial for ICP control both alone and where EVD has failed. Although the evidence base is insufficient to draw firm conclusions, based on the available evidence, there is no clear indication that the complication rates of ELD are greater than those of EVD. Further large prospective observational studies are required to generate sufficient support for an acceptable safety profile, with the possibility of subsequent randomised controlled studies to ultimately assess therapeutic parity.
